# Keratin 17 and Collagen type 1 genes: Esophageal cancer molecular marker discovery and evaluation

**DOI:** 10.1111/crj.13793

**Published:** 2024-07-09

**Authors:** Huiwen Pan, Jie Hong, Aizhong Shao, Zhiguo Zhao, Guowen Ding, Zhijie Fang, Keping Chen, Jingfeng Zhu

**Affiliations:** ^1^ School of Food and Biological Engineering Jiangsu University Zhenjiang China; ^2^ The Affiliated People's Hospital of Jiangsu University Zhenjiang China; ^3^ Department of Otolaryngology The Affiliated Suzhou Hospital of Nanjing Medical University, Suzhou Municipal Hospital, Gusu School, Nanjing Medical University Suzou China

**Keywords:** COL1A1, combined detection, esophageal cancer, KRT17, marker

## Abstract

One hundred eighty pairs of tissues of esophageal squamous cell carcinoma (ESCC) were tested by the transcriptome sequencing in order to explore etiology factors. The chi‐square test and correlation analysis demonstrated that the relative expression levels of keratin 17 (KRT17) and collagen type I α1 chain (COL1A1) were significantly higher in EC with diabetes. Expression of KRT17 was correlated with blood glucose (*r* = 0.204, *p* = 0.001) and tumor size (*r* = −0.177, *p* = 0.038) in patients. COL1A1 correlated with age (*r* = −0.170, *p* = 0.029) and blood glucose levels (*r* = 0.190, *p* = 0.015). Experimental results of qRT‐PCR: KRT17 and COL1A1 genes were highly expressed in ESCC (*p* < 0.05). When the two genes were used as a combination test, the positive detection rate of EC was 90.6%, and the ROC curve had greater power. The KRT17 and COL1A1 genes had the potential to be biomarkers for the diagnosis of ESCC.

## Graphical abstract

Testing the KRT17 gene and the COL1A1 gene from 180 paired ESCC tissues by using transcriptome technology, para‐cacinoma tissues as a control group and carcinoma tissues as a case group. Recording every factor, including patient sex, age, smoking, alcohol, blood glucose, and tumor size, from the electronic medical system. Analyzing experimental data from two groups, both KRT17 and COL1A1 mRNA were highly expressed in case tissues, which are also correlated with age, blood glucose and tumor size. KRT17 and COL1A1 are potential biomarkers for esophageal cancer in the Chinese population.

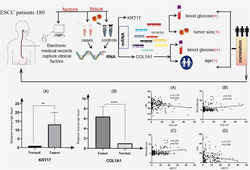

**Abbreviations**
ESCC: esophageal squamous cell carcinoma
EAC: esophageal adenocarcinoma
WGS: whole genome sequencing
COL1A1: collagen type I α1 chain
KRT17: keratin 17
PCR: polymerase chain reaction
OR: odds ratio

## 1 INTRODUCTION

**FIGURE 1:**
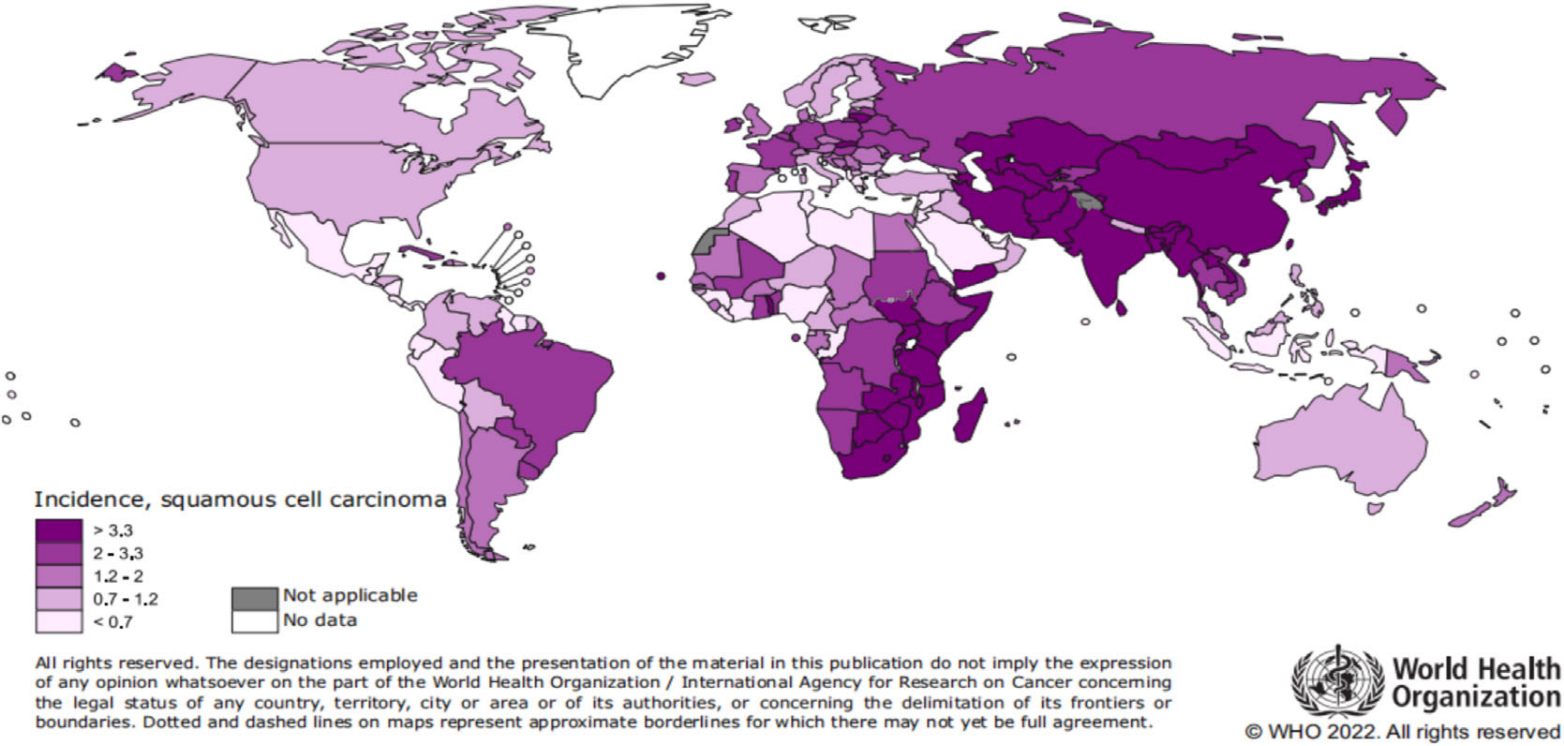
Incidence profile of esophageal squamous cell carcinoma worldwide during 2022 from World Health Organization (WHO). https://www.cancer.gov/types/esophageal.

Esophageal cancer (EC) is the eighth most common cancer and the sixth most common cause of cancer death worldwide.^1^ According to the type of malignant cells, EC has two different pathological types: adenocarcinoma and squamous cell carcinoma.^2^ According to the World Health Organization report in 2022, the incidence of squamous cell carcinoma is most severe in Asia, especially in China, accounting for more than half of EC cases worldwide (Figure 1). Esophageal squamous cell carcinoma (ESCC) accounts for approximately 90% of cases worldwide and is mainly esophageal carcinoma in China.^3^, ^4^ The early clinical symptoms of EC are not obvious, patients in the advanced stage are even unable to swallow water with a poor prognosis 5‐year survival rate less than 20%. Therefore, it is urgent to explore the molecular mechanisms and find screening therapeutic targets and prognostic markers.^5^

The treatment methods for EC mainly include surgery, radiotherapy, chemotherapy,^5^ targeted therapy,^6^, ^7^ and immunotherapy^8^ clinically, but with great trauma and poor recovery. Studies have shown that long‐term smoking,^9^ alcohol consumption,^10^, ^11^ blanching diet and the contents of some vitamins,^12^, ^13^ minerals, trace elements,^14^, ^15^ mildewed food, dietary patterns^16^ are high‐risk factors.^17^, ^18^ Genetic studies still lack specific screening indicators, although studies have preliminarily shown that genes are associated with EC. For example, Li‐Yan Guo^19^ showed that the PLCE1 gene interacted with environmental factors and that the occurrence of ESCC and rs227423, a polymorphism of the PLCE1 gene, played a role in the ESCC population with family history. Cheng^20^ tested the feasibility of epidermal growth factor receptor (EGFR) gene‐targeted chimeric antigen receptor T‐cell immunotherapy (CAR‐T) cells for the treatment of ESCC in a large Chinese sample by whole genome sequencing (WGS) and RNA‐seq analysis. A circRNA study^21^ found that the cirbe4b‐mitogen‐activated protein kinases/extracellular signal‐regulated kinase (MAPK/ERK) signaling pathway could potentially be a biological target for ESCC therapy.

The collagen type I α1 chain (COL1A1) gene belongs to the collagen family and is involved in epithelial–mesenchymal transition (EMT). Studies^22^, ^23^ have shown that diseases associated with COL1A1 also include Caffey's disease and type I osteogenesis imperfecta disease. In recent years, COL1A1 has been shown to be highly expressed in a variety of cancers.^22^, ^23^, ^24^, ^25^ A recent review of biomarkers and therapeutic targets for chondrosarcoma has also summarized several promising gene targets, including COL1A1 and COL2A1.^2^, ^26^, ^27^, ^28^, ^29^, ^30^

**FIGURE 2:**
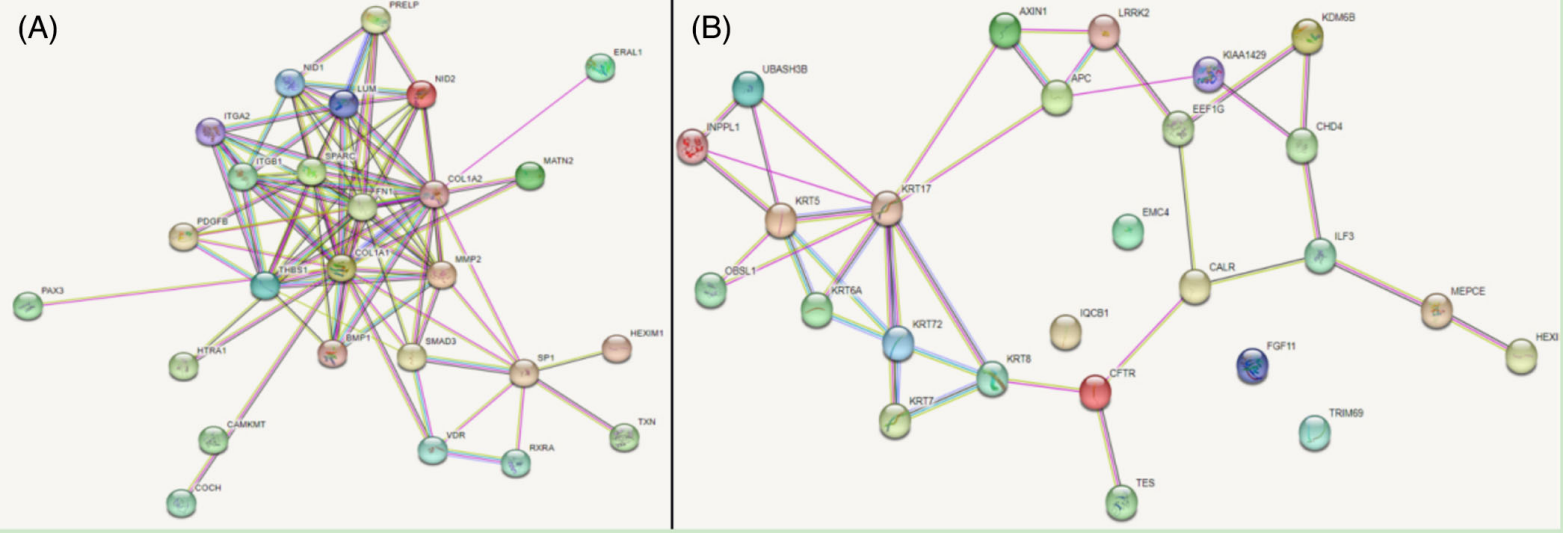
Collagen type I α1 chain (COL1A1) or keratin 17 (KRT17) gene STRING Interaction Network Preview. (A) COL1A1 gene. STRING Interaction Network Preview; (B) KRT17 gene STRING Interaction Network Preview. From Genecards online network.

The COL1A1 gene and keratin 17 (KRT17) network pathway played a role (Figure 2). KRT17 is predominantly expressed in the appendages of epidermal tissues. EC is a kind of epithelial tumor. KRT17 and COL1A1 were associated with cancers, such as DNA damage response^30^ and the progression of conventional renal cell carcinoma and a low expression in poor prognosis of bladder cancer.^31^, ^32^, ^33^ However, no study has been conducted on the combined effect of COL1A1 and KRT17 in the Chinese ESCC population. Based on this, this study was performed.

## 2 MATERIALS AND METHODS

### 2.1 Specimen and clinical information collection

The study had the informed consent of the participants. All the experiments were in accordance with the Declaration of Helsinki. This study was approved by the Ethics Committee of the Affiliated People's Hospital of Jiangsu University.

Between 2018 and 2020, 180 pairs of ESCC surgical pathological tissues were collected from the Affiliated People's Hospital of Jiangsu University. The cancer tissues were the case group, and the adjacent normal tissues were the control group. The surgical tissue was immediately placed in a cryotube with an RNA tissue preservation solution and stored in liquid nitrogen.

We collected personal data from questionnaires, medical records, and interviews and recorded the required information, such as smoking, drinking, blood sugar, and other indicators, in Excel tables. Clinical information includes recording gender, age, smoking and drinking habits, fasting blood glucose, past medical history of hypertension and diabetes, histological tissue differentiation degree, and tumor size.

### 2.2 Exclusion criteria

Request is primary ESCC in Chinese population. Non‐ESCC were excluded. Exclude patients with other cancers that have metastasized to EC. Patients who had undergone treatments prior to surgery were excluded.

### 2.3 Extraction of total RNA

A total of 180 pairs of RNA were extracted by the TRIzol method, and genomic DNA was removed by DNase I. The quality of RNA samples was detected by 2100 Bioanalyser and ND‐2000 methods to ensure that qualified samples (OD260/280 = 1.8–2.2, OD260/230 ≥ 2.0, RNA integrity number [RIN] ≥ 6.5, 28S: 18S ≥ 1.0, > 5 μg) were used for transcriptome sequencing.

### 2.4 Transcriptome sequencing

Raw data stored in fastq format were obtained using the Illumina Hiseq sequencing platform. First, fastp was used to evaluate the sequencing‐related quality of the original sequencing data, and the base quality, base error rate, and base content distribution were analyzed to obtain high‐quality quality control data (clean data), ensuring the accuracy of the results. Subsequently, the high‐quality de‐adaptor reads data were compared with the human GRCh38.p13 reference genome using the improved BWT algorithm (http://asia.ensembl.org/Homo_sapiens/Info/Index). Finally, the software StringTie (http://ccb.jhu.edu/software/stringtie/) was used to assemble the mapped reads by reference‐based assembly.

### 2.5 Screening for differentially expressed genes

After obtaining the read counts for genes/transcripts, DESeq2 was employed to perform differential gene expression analysis between multiple samples. This analysis identified differentially expressed transcripts. To obtain significantly different genes, the filtering conditions were set as follows: *p*‐value < 0.05 and |FoldChange| > 2.

### 2.6 Q‐PCR validation of differentially expressed genes

The selected differentially expressed genes, KRT17 and COL1A1, were selected for large sample validation, and 180 pairs of tissues from patients were used, and there was no significant difference in age and gender between the two groups with comparability. Total RNA was extracted from the samples by the TRIzol method, and then the extracted total RNA was reverse transcribed into cDNA using HiScript III RT SuperMix for qPCR (+gDNA wiper), a reverse transcription kit produced by Vazyme, China, and reverse transcribed on a PCR instrument according to the following reaction conditions:

Reaction system and conditions: RNase‐free ddH2O, 16 μL, 4 × gDNA wiper Mix, 4 μL, Template RNA 1 μg, 4 μL 5 × HiScript III qRT SuperMix, 16 μL 1st reaction solution reaction condition: 42°C, 2 min. Reaction conditions: 37°C for 15 min, 85°C for 5 s, −20°C for short‐term storage, −70°C for long‐term storage.

Primer design for the KRT17 and COL1A1 genes was carried out using Primer 5.0 software, as shown in Table 1, and detailed specificity prediction was performed using Primer‐BLAST software. The primers were synthesized by Shanghai Biotechnology Co., Ltd.

**TABLE 1:** Primers were designed for KRT17 and COL1A1 genes.

Gene	Primer	Sequence	Number
KRT17	Upstream primer	GGAGCAGCAGAACCAGGAAT	SEQ ID NO.1
	Downstream primer	GGTCACCGGTTCTTTCT	SEQ ID NO.2
COL1A1	Upstream primer	GCCAAGACGAAGACATCCCA	SEQ ID NO.3
	Downstream primer	GGCAGTTCTTGGTCTCGTCA	SEQ ID NO.4

Abbreviations: COL1A1, collagen type I α1 chain; KRT17, keratin 17.

### 2.7 Real‐time fluorescence quantitative PCR detection of KRT17 and COL1A1 gene expression

Quantitative gene expression analysis was performed using the AceQ Universal SYBR qPCR Master Mix fluorescence quantitative reagent kit from Vazyme. The cDNA concentration was measured and diluted using Nanodrop 2000, and the diluted cDNA (100 ng/μL) was used as the template for RT‐PCR. The reaction system and program are as follows (Tables 2 and 3):

**TABLE 2:** qRT‐PCR reaction system.

Reagent	Volume
2 × AceQ Universal SYBR qPCR Master Mix	10.0 μL
Forward primer (10 μM)	0.4 μL
Reverse primer (10 μM)	0.4 μL
Template cDNA	100 ng/1 μL
ddH2O	To 20 μL

**TABLE 3:** qRT‐PCR reaction procedure.

Stage	Cycle	Temperature (°C)	Time
Predenaturation	1 min	95°C	5 min
Cyclic reaction	40 s	95°C	10 s
		60°C	30 s
Dissolution curve	1 min	95°C	15 s
		60°C	60 s
		95°C	15 s

Quantitative expression of genes was detected using AceQ Universal SYBR qPCR Master Mix, a fluorescence quantitative kit for Vazyme. The cDNA concentration was measured and diluted using Nanodrop 2000, and the diluted cDNA (100 ng/μL) was used as the template for RT‐PCR. Each RNA template was tested in triplicate. The reaction system and reaction procedure are as follows (Tables 2 and 3).

### 2.8 Statistical methods

The result data were visualized as bar graphs using GraphPad Prism 8 software, represented as geometric means ± standard deviation. Statistical analysis was performed using SPSS 23.0 software, and the differences between the two were assessed using a *t*‐test. The chi‐square test and Bonferroni test were from SPSS 20.0 software.

## 3 RESULTS

### 3.1 Chi‐square test for the relative expression levels of KRT17 and COL1A1

Bold diabetes in Table 4: in the KRT17‐RQ lower group, ESCC had diabetes mellitus of 1.61% (number = 1); however, they had diabetes mellitus of 10.26% (number = 12) in the KRT17‐RQ higher group. The difference was statistically significant (*p* = 0.036) and the Bonferroni test was 0.047.

**TABLE 4:** Chi‐square test of clinical factors for KRT17 and COL1A1 relative gene expression and dichotomous data.

Factors		KRT17‐RQ		*χ* ^2^	*p*	COL1A1‐RQ		*χ* ^2^	*p*
		Lower^a^ (%)	Higher (%)			Lower^a^ (%)	Higher (%)		
Sex	Male	47 (75.81)	88 (75.21)			34 (70.83)	101 (71.63)		
	Female	15 (24.16)	29 (24.79)	0.008	0.930	14 (29.17)	30 (28.37)	0.744	0.388
Age	≤66	28 (45.16)	51 (43.59)			19 (39.58)	60 (42.56)		
	>66	34 (54.84)	66 (56.41)	0.041	0.840	29 (60.42)	71 (57.44)	0.551	0.458
Tobacco	−	34 (54.84)	72 (61.54)			29 (60.42)	77 (58.78)		
	+	28 (45.16)	45 (38.46)	0.753	0.385	19 (39.58)	54 (41.22)	0.039	0.843
Alcohol	−	37 (59.68)	79 (67.52)			28 (60.87)	71 (61.21)		
	+	25 (40.32)	38 (32.48)	1.093	0.296	18 (39.13)	45 (38.80)	0.002	0.968
Hypertension	−	46 (74.19)	76 (64.96)			34 (70.83)	88 (67.18)		
	+	16 (25.80)	41 (35.04)	1.593	0.240	14 (29.17)	43 (32.82)	0.617	0.214
**Diabetes**	**−**	61 (98.39)	105 (89.74)			47 (97.92)	119 (90.84)		
	**+**	1 (1.61)	12 (10.26)	4.495	**0.036**	1 (2.08)	12 (9.16)	2.612	0.190
Lymphaden	−	34 (55.74)	69 (58.97)			24 (63.16)	79 (60.76)		
	+	27 (44.26)	48 (41.03)	0.172	0.678	14 (36.84)	51 (39.23)	0.071	0.790
BMI	<24	45 (73.77)	81 (69.23)			33 (68.75)	93 (71.54)		
	≥24	16 (26.23)	36 (30.77)	0.400	0.527	15 (31.25)	37 (28.46)	0.132	0.717

*Note*: *p* < 0.05, representing a statistically significant difference. “+” indicates positive, and “−” indicates negative.

Abbreviations: COL1A1, collagen type I α1 chain; KRT17, keratin 17; RQ, relative quantity.

^a^ Lower, relatively low expression group when RQ value ≤ 2.

### 3.2 Relative expression of KRT17 and COL1A1 and quantitative indicators of clinical factors

### 3.3 Transcriptome sequencing results

**FIGURE 3:**
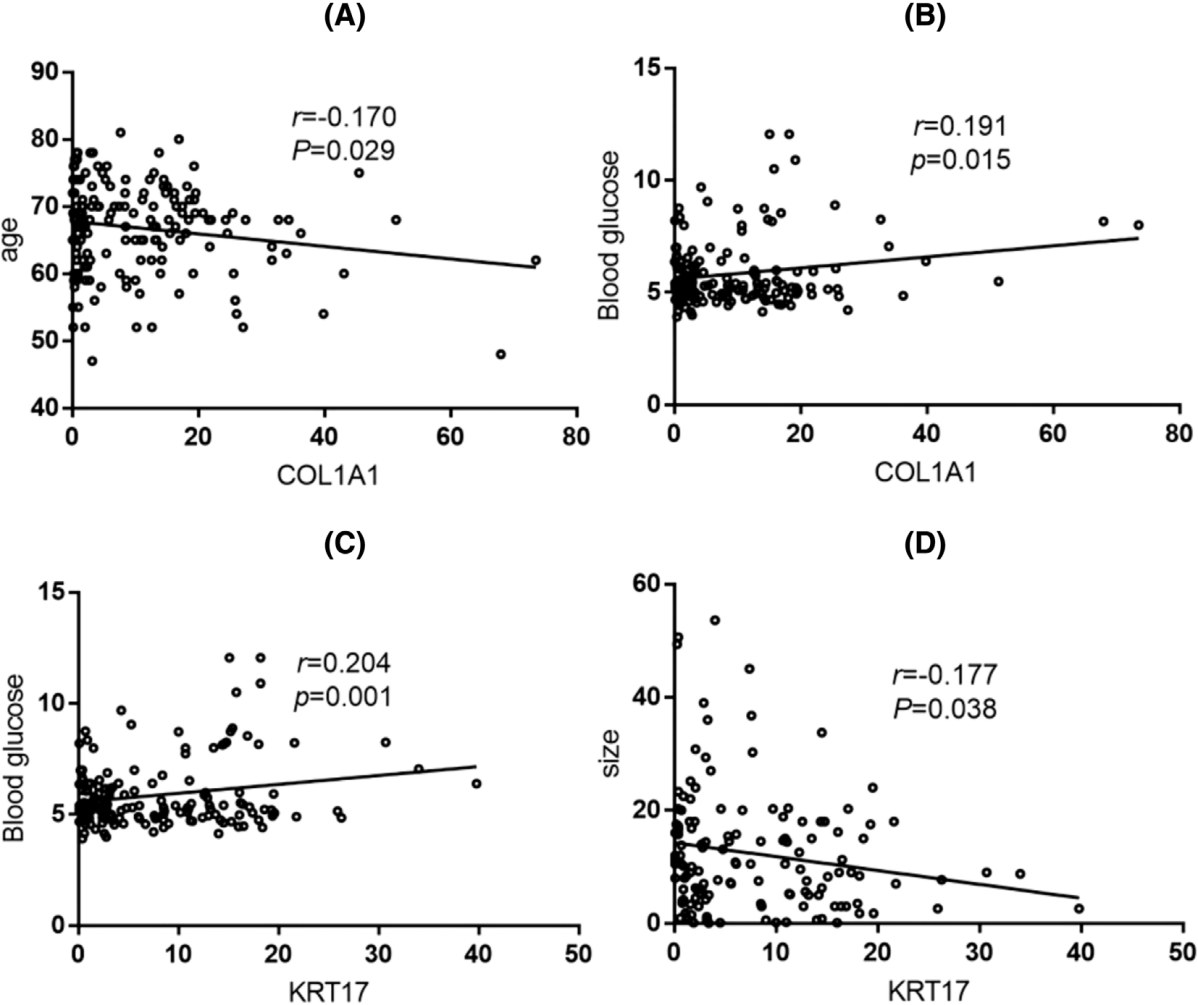
Correlation analysis between keratin 17 (KRT17) and collagen type I α1 chain (COL1A1) genes relative expression and clinical factors.

The relative expression of COL1A1 mRNA was correlated with the age of patients (*r* = −0.170, *p* = 0.029, see Figure 3A). The relative expression level of COL1A1 mRNA was positively correlated with the blood glucose of patients (*r* = 0.191, *p* = 0.015, see Figure 3B). The relative expression level of KRT17 mRNA was positively correlated with the blood glucose of patients (*r* = 0.204, *p* = 0.001, see Figure 3C). KRT17 mRNA was positively correlated with the size of the tumor (*r* = 0.204, *p* = 0.001, see Figure 3D).

#### 3.3.1 Quality testing of transcriptome sequencing samples

**FIGURE 4:**
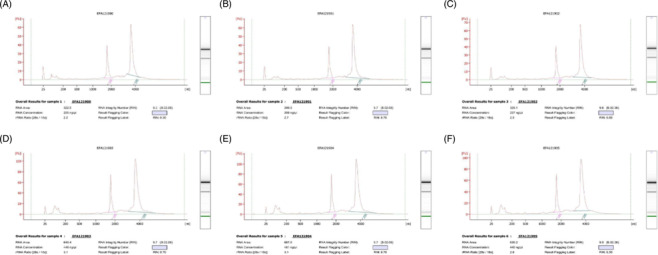
Quality detection map of transcriptome sequencing samples. A, B, C: tumor; D, E, F: control.

Figure 4, nucleic acid running gel was performed on the extracted RNA experiment, and its RIN value and optical density (OD) ratio were determined, showing that the sequenced RNA was of good quality.

#### 3.3.2 Expression of transcriptome sequencing differential genes KRT17 and COL1A1 in tumor and normal tissues

**FIGURE 5:**
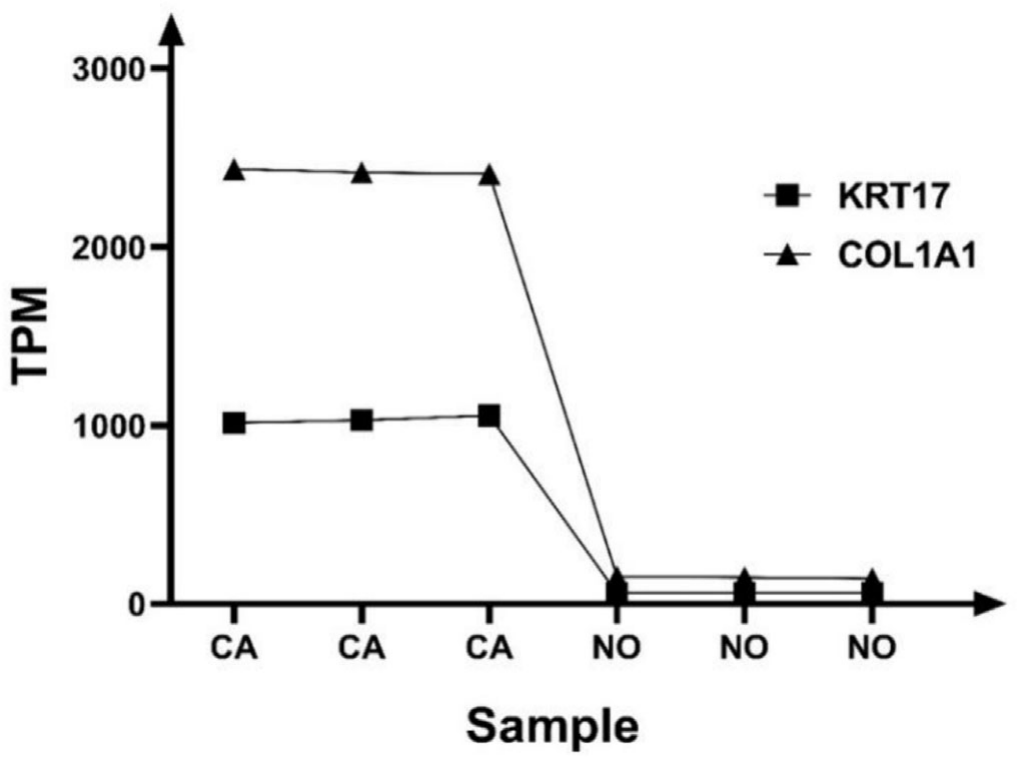
Expression levels of differential genes keratin 17 (KRT17) and collagen type I α1 chain (COL1A1) in tumor and control groups. CA, cancer group; NO, control group; TPM, transcripts per million.

Figure 5, KRT17 and COL1A1 genes were selected as the highly expressed significantly differential genes, which are closely related to ESCC. KRT17 and COL1A1 were screened based on expression level, fold difference, *p* < 0.05, and enrichment analysis.

### 3.4 Differential expression of KRT17 and COL1A1 genes in tumor and normal tissues detected by real‐time PCR

Individual real‐time fluorescence quantification showed that KRT17 gene expression levels were significantly increased in tumor tissues from 145/180 patients with EC, and COL1A1 gene expression levels were significantly increased in tumor tissues from 152/180 patients.

**FIGURE 6:**
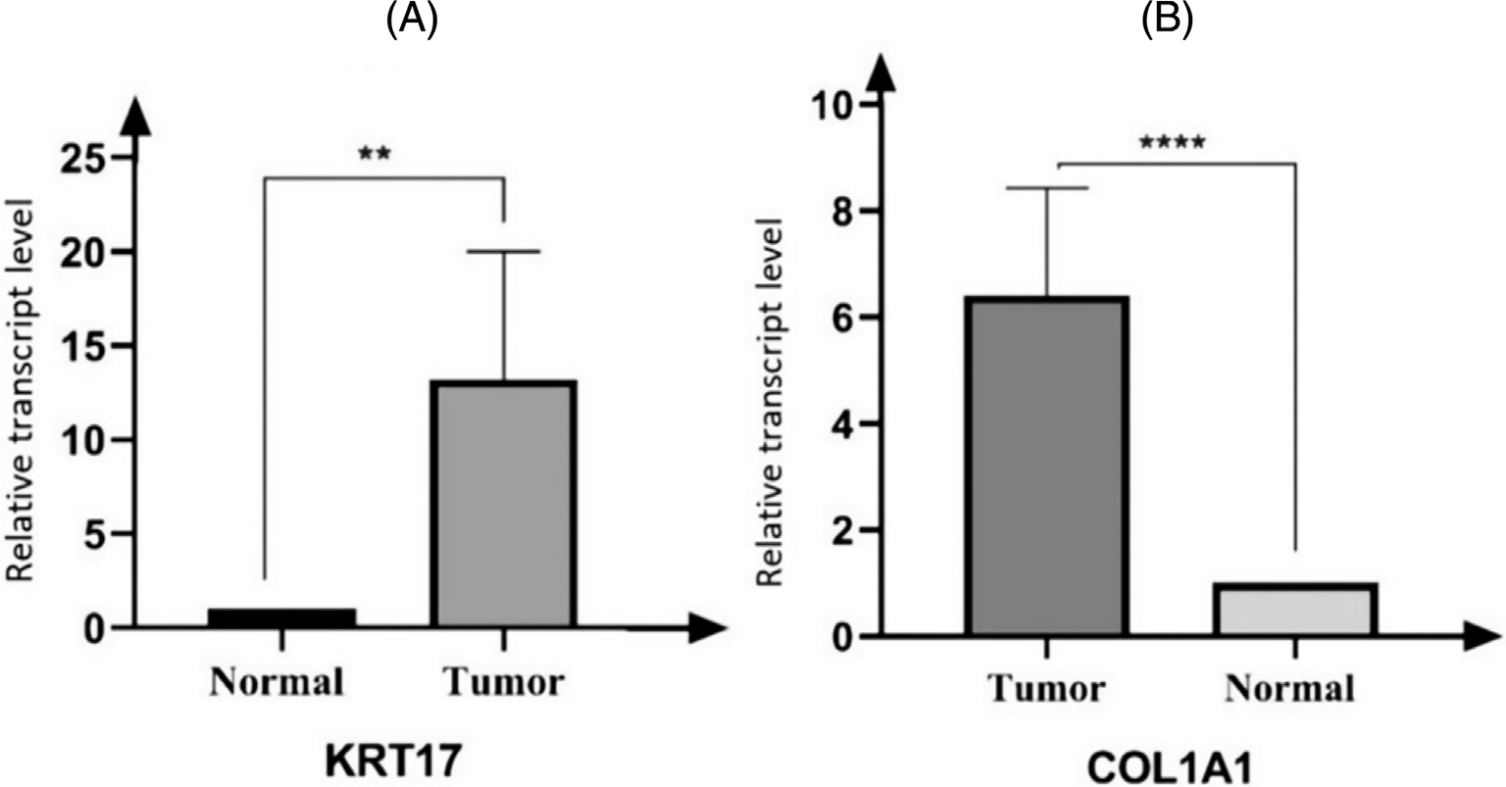
qRT‐PCR detecting the difference in gene expression between the two groups. Vertical axis: relative transcript level − relative expression level. Horizontal axis: sample groups (normal group and cancer tissue group). ** is *p* < 0.01; **** is *p* < 0.001.

Results are shown in Figure 5A,B; the relative expression levels of the KRT17 and COL1A1 genes were significantly higher in tumors than normal, and the difference was statistically significant (*p* < 0.05) (Figure 6).

### 3.5 ROC curve analysis

To assess the potential power of KRT17 and COL1A1 as diagnostic markers for EC, receiver operating characteristic curves (ROC) were used for analysis.

**FIGURE 7:**
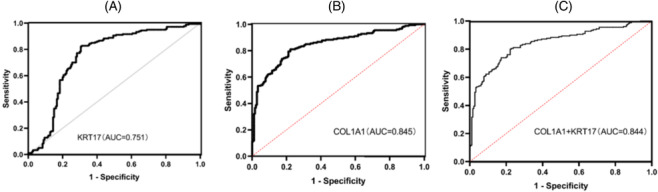
Receiver operating characteristic curves (ROC) for keratin 17 (KRT17) and collagen type I α1 chain (COL1A1). (A) KRT17 individual ROC curve profile plot. (B) Characteristic plot of COL1A1 separate ROC curve. (C) ROC curve characteristic plot for combined KRT17 + COL1A1 test.

As shown in Figure 7A–C, the area under curve (AUC) values of KRT17 and COL1A1 for EC were found to be 0.751, 0.845, and 0.844, respectively. The AUC values of all three were ≥0.7 and when COL1A1 and KRT17 were used in combination ≥0.8, which had some power in the test.

The area under the ROC curve was greater than 0.5 (AUC > 0.5), representing that these two genes alone or in combination have reference values for detection efficacy (Table 5).

**TABLE 5:** Core genes KRT17 and COL1A1 were used to test the efficacy of esophageal cancer.

Gene	AUC	Cut‐off	Sensitivity	Specificity
KRT17	0.751	4.148	0.828	0.689
COL1A1	0.845	1.053	0.806	1.000

Abbreviations: COL1A1, collagen type I α1 chain; KRT17, keratin 17.

**FIGURE 8:**
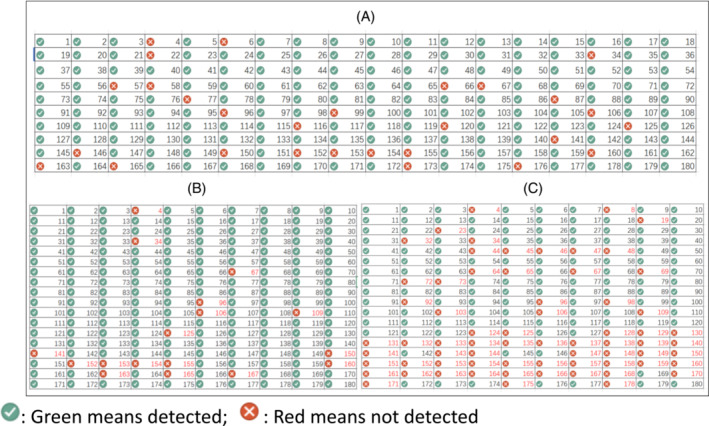
Positive rates of esophageal cancer were detected separately and jointly when keratin 17 (KRT17) and collagen type I α1 chain (COL1A1) were highly expressed. 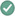: Green means detected; 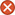: Red means not detected. (A) COL1A1 positive rate of 84.4% (152/180). (B) COL1A1 + KRT17 positive rate of 90.6% (163/180). (C) KRT17 positive rate of 62.8% (113/180).

Figure 8, alone COL1A1 positive was found is 152/180 in EC patients, with a detection rate of 84.4%; alone KRT17 positive was found is 113/180 in esophageal patients, with a detection rate of 62.8%; and 163/180 positive patients were found when combined use of these two genes (COL1A1 + KRT17, the same time), with a high rate of 90.6%, which could better reduce the possibility of detection errors caused by the use of the two alone.

## 4 DISCUSSION

COL1A1 is highly expressed in a variety of cancers and regulates cellular processes, and high COL1A1 expression is also associated with poor cancer prognosis.^5^ A study based on animal observations in mice and computer imaging systems from the University of Michigan and other institutions has found that when researchers eliminate COL1A1 production from tumor cells, animal models with brain tumors may live longer,^28^ which showed COL1A1 as a feasible target to disrupt tumor progression in the latest spatiotemporal analysis study of glioma heterogeneity.

Another study from Huang et al.^34^ researchers detected the expression of FBXL19‐AS1, miR‐193a‐5p, and COL1A1 by RT‐PCR and western blot and determined the proliferation, migration, invasion, apoptosis, and EMT of cervical cancer cells. The results showed that high COL1A1 expression was associated with the proliferation and metastasis of cervical cancer. FBXL19‐AS1 promotes the proliferation and metastasis of cervical cancer cells by sponging miR‐193a‐5p and up‐regulating COL1A1.

Meanwhile, KRT17 played a role in DNA damage response and tumor initiation.^8^, ^9^ High levels of KRT17 are associated with poor prognosis in several human cancers. Raji R.^31^experimented through mouse studies and cell comparison research showed that KRT17 high expression is positively significant with skin cancer growth between the test group and control, a lack of KRT17 delays tumor proliferation, and high levels of KRT17 expression are not conducive to the prognosis of the disease.

The similarities between this study and many of the above studies suggest that these two genes are expressed at high levels in ESCC. Although both KRT17 and COL1A1 expression levels are highly expressed in ESCC tissues, they vary according to the specific population. Relative expression of KRT17 and COL1A1 was both higher in ESCC patients with diabetes and correlated with blood glucose levels. In a nut word, those ESCC patients with high blood glucose values had higher KRT17 and COL1A1 expression levels. Research suggests that there may be an intrinsic link between high and low sugar levels in ESCC. When it comes to sugar and cancer research, researchers Hedong Han et al.^35^ explored the relationship between blood glucose concentration and the risk of liver cancer. They conducted a meta‐analysis of prospective studies by using a database of PubMed, EMBASE, and the Cochrane Library through October 2016 with a total of 1975 liver cancer cases. This research revealed that blood glucose increases the risk of liver cancer across the range of prediabetes and diabetes. In the next research plan, we will demonstrate the relationship between sugar and EC through meta‐analysis.

On the other hand, it was negatively correlated with tumor size; that is, the smaller the tumor, the higher the KRT17 expression level; otherwise, the KRT17 expression level decreased as the tumor grew larger. It suggests that this gene is significant in the early stages of a tumor, and the KRT17 screening test should be performed early when the tumor is small so that it can be detected as soon as possible.

COL1A1 expression is negatively correlated with age (lower age corresponds to higher COL1A1 expression in patients) and positively correlated with blood glucose level (higher blood glucose, higher COL1A1 expression). All these suggest that targeted screening for COL1A1 expression should be focused on individuals of younger age (<66 years), and at the same time, those esophageal tumor patients with hyperglycemia or diabetes should be strengthened for COL1A1 gene screening.

Even so, we have to admit that there are several limitations to this research. The primary limitation of this study is the relatively small sample size, especially for the controls and ESCC group. However, this study undoubtedly shows a significant association between the KRT17 and COL1A1 genes, of course, this association need a larger ESCC sample. The findings of this single‐center study would be strengthened by corroboration from other larger studies. The other differences in cell experiments were not included in the current study, and their inclusion in future research may expand the role of these two genes in ESCC and prognosis.

Our study is still ongoing with the postoperative follow‐up of EC patients. Survival indicators for 5‐year prognoses will be collected. We will conduct in‐depth analyses, considering the expression patterns of the KRT17 gene and COL1A1, to assess their impact on both the prognosis of patients survival time and quality of life. Targeted follow‐up studies on KRT17 and COL1A1 genes with ESCC survival may help not only elucidate the mechanisms that relate the two genes to tumor initiation and, more broadly, the roles of KRT17 and COL1A1 during tumorigenesis but also understand how these elements play out in different populations of ESCC and other tissues.

## AUTHOR CONTRIBUTIONS

Huiwen Pan, Zhijie Fang, Jingfeng Zhu, and Keping Chen made contributions to the conception, design, conduct, and data acquisition. Huiwen Pan, Aizhong Shao, Guowen Ding, Zhiguo Zhao, and Jie Hong interpreted the clinical trial, samples, experiments, data, and interpretation. Huiwen Pan wrote and edited the manuscript. All authors agreed to be personally accountable for their own contributions.

## CONFLICT OF INTEREST STATEMENT

The authors declare that they have no competing interests.

## ETHICS APPROVAL

All the experiments in this study were conducted in accordance with the relevant guidelines and regulations and in accordance with the Declaration of Helsinki. This study was approved by the Ethics Committee of the Affiliated People's Hospital of Jiangsu University.

## Supporting information


**Data S1.** Supplementary Information.

## Data Availability

The authors confirm that the data supporting the findings of this study are available within the article and its supplementary materials.
